# Supplementary material for: A Flexible Approach for Assessing Heterogeneity of Causal Treatment Effects on Patient Survival Using Large Datasets with Clustered Observations
Source: Int J Environ Res Public Health. 2022 Nov 12;19(22):14903. doi: 10.3390/ijerph192214903 (PMC9690785; doi:10.3390/ijerph192214903)
Supplement: Supplementary file 1 [file ijerph-19-14903-s001.zip › ijerph-1936020-supplementary.pdf]

Supplemental Materials for "A flexible approach for assessing heterogeneity of causal treatment effects on patient survival using large datasets with clustered observations by Hu et al."

S1. The proposed method riAFT-BART

We consider a two-level data structure that has K clusters, each having n_k individuals, indexed by $i = 1, \dots, n_k, k = 1, \dots, K$. The total sample size is $N = \sum_{k=1}^{K} n_k$. There can be multiple treatments, denoted by $A \in \mathcal{A} = \{ a_1, \dots, a_J\}$. For each individual i in cluster k, there is a vector of pre-treatment measured covariates X_{ik}, and let T_{ik} be the individual's failure time, which may be right censored at C_{ik}. Let V_k be the cluster indicators. We estimate the causal effect in the potential outcomes framework, and denote the potential (counterfactual) survival time under treatment a_j using $T_{ik}(a_j)$, for $j = 1, \dots, J$. We can define a pairwise treatment effect between a_j and $a_{j'}$ on patient survival by contrasting the counterfactual survival times under the two treatments averaged over a population or a subpopulation.

To estimate the treatment effects from observed data, we maintain the standard causal assumptions for clustered survival data, and propose the following random-intercept accelerated failure time model with Bayesian additive regression trees (riAFT-BART),

$$\log T_{ik} = f(A_{ik}, X_{ik}) + b_k + \epsilon_{ik},$$
$$b_k \sim N(0, \alpha_k \tau^2), \epsilon_{ik} \sim N(0, \sigma^2), b_k \perp \epsilon_{ik},$$

where $f(A_{ik}, X_{ik})$ is an unspecified function relating treatments and covariates to survival times, b_k's are the random intercepts for cluster-specific main effects, and ϵ_{ik} is the residual term. We use Bayesian machine learning BART to flexibly model the unknown function f, and assume a mean-zero normal distribution for and independence between b_k and ϵ_{ik} with variance $\alpha_k \tau^2$ and σ^2, respectively. We place priors on the model parameters and develop an efficient Markov chain Monte Carlo (MCMC) algorithm to draw posterior distributions of the parameters, which can be used to draw posterior inferences about the survival treatment effects. The counterfactual survival curve can be derived corresponding to our riAFT-BART model as $P(T_{ik} > t \mid A_{ik}, X_{ik}, \sigma, b_k) = 1 - \Phi(\frac{\log t - f(A_{ik}, X_{ik}) - b_k}{\sigma})$, which can be used as the basis to objectively compare the proposed method with other approaches (see S2. Simulation design).

Complete details for the development of the riAFT-BART model and MCMC algorithm, as well as the estimation of population average treatment effects via riAFT-BART model can be found in our statistical methodology work.[1] In this article, we use riAFT-BART to evaluate the heterogenous treatment effects on patient survival.

S2. Simulation design

The simulation was designed to generate datasets that represented the characteristics possessed by the NCDB data; and to make it difficult for any method to successfully estimate the true treatment effects. To assess the comparative performance of our proposed method, we adapted the popularly used inverse probability weighting method into the setting of clustered and censored survival data to form two comparison methods: inverse probability of treatment weighting with the random-intercept Cox regression model (IPW-riCox) and doubly robust random-intercept additive hazards model (DR-riAH).[2] In addition, we consider another outcome modeling based method, the random-intercept generalized additive proportional hazards model (riGAPH),[3] that is flexible at capturing nonlinear relationships. We use the counterfactual survival curve as the basis to objectively compare methods. The performance metrics used to evaluate the methods were the bias, root-mean-squared-error (RMSE) and precision in the estimation of heterogeneous effects (PEHE) in the estimation of the heterogeneous treatment effects. Both survival probability at a fixed time point (e.g. 5-year) and the conditional restricted mean survival time (RMST) [4] were used to estimate the treatment effects.

Following the recommended strategies[5,6] for assessing the performance of methods in estimating heterogeneous treatment effects, we subclassified the simulated individuals into 45 subgroups based on the distribution of the true generalized propensity scores, and calculated the bias and RMSE for each method and for each propensity score subgroup across 250 data replications. The PEHE measures the difference between the true and estimated survival probabilities or RMST across all data points, and a smaller value of PEHE indicates better accuracy and is considered favorable. The detailed subclasses of generalized propensity scores are presented in Table S1. Note that the generalized propensity scores for each individual sum to one, $P(A_{ik} = 1 \mid X_{ik}, b_k) + P(A_{ik} = 2 \mid X_{ik}, b_k) + P(A_{ik} = 3 \mid X_{ik}, b_k) = 1$. The subclassification is based on intervals of the true propensity scores for treatment 1 and treatment 2, representing a full range

of assignment propensity to each treatment group. The centroid region of the propensity score distribution includes individuals at clinical equipoise (and for whom the treatment decisions are mostly unclear) and resemble those recruited in a randomized controlled trial. In our simulation, subclasses 8-12 (Table S1) represent the centroid region.

Table S1: Subclasses of the true generalized propensity scores

Subclass ID	True propensity score for treatment group 1	True propensity score for treatment group 2
**1**	(0, 0.05]	(0.95, 1]
**2**	(0, 0.25]	(0, 0.5]
**3**	(0, 0.25]	(0.5, 0.75]
**4**	(0, 0.25]	(0.75, 1]
**5**	(0.25, 0.5]	(0, 0.25]
**6**	(0.25, 0.3]	(0.65, 0.7]
**7**	(0.25, 0.3]	(0.7, 0.75]
**8**	(0.25, 0.5]	(0.25, 0.3]
**9**	(0.25, 0.5]	(0.3, 0.35]
**10**	(0.25, 0.5]	(0.35, 0.4]
**11**	(0.25, 0.5]	(0.4, 0.45]
**12**	(0.25, 0.5]	(0.45, 0.5]
**13**	(0.25, 0.5]	(0.5, 0.55]
**14**	(0.25, 0.5]	(0.55, 0.6]
**15**	(0.3, 0.35]	(0.6, 0.65]
**16**	(0.3, 0.35]	(0.65, 0.7]
**17**	(0.35, 0.4]	(0.6, 0.65]
**18**	(0.5, 0.55]	(0.25, 0.3]
**19**	(0.5, 0.55]	(0.3, 0.35]
**20**	(0.5, 0.55]	(0.35, 0.4]
**21**	(0.5, 0.55]	(0.4, 0.45]
**22**	(0.5, 0.55]	(0.45, 0.5]
**23**	(0.55, 0.6]	(0.25, 0.3]

**24**	(0.55, 0.6]	(0.3, 0.35]
**25**	(0.55, 0.6]	(0.35, 0.4]
**26**	(0.55, 0.6]	(0.4, 0.45]
**27**	(0.6, 0.65]	(0.2, 0.25]
**28**	(0.6, 0.65]	(0.25, 0.3]
**29**	(0.6, 0.65]	(0.3, 0.35]
**30**	(0.6, 0.65]	(0.35, 0.4]
**31**	(0.65, 0.7]	(0.2, 0.25]
**32**	(0.65, 0.7]	(0.25, 0.3]
**33**	(0.65, 0.7]	(0.3, 0.35]
**34**	(0.7, 0.75]	(0.2, 0.25]
**35**	(0.7, 0.75]	(0.25, 0.3]
**36**	(0.5, 0.75]	(0, 0.15]
**37**	(0.5, 0.75]	(0.15, 0.2]
**38**	(0.75, 0.8]	(0.15, 0.2]
**39**	(0.75, 0.8]	(0.2, 0.25]
**40**	(0.8, 0.85]	(0.1, 0.15]
**41**	(0.8, 0.85]	(0.15, 0.2]
**42**	(0.85, 0.9]	(0.1, 0.15]
**43**	(0.9, 0.95]	(0.05, 0.1]
**44**	(0.75, 1]	(0, 0.05]
**45**	(0.75, 1]	(0.05, 0.1]

We generated $K = 20$ clusters, each with a sample size of $n_k = 850$. The total sample size is $N = 17000$. We simulated 10 confounders, with five continuous variables independently generated from the standard normal distribution $X_{ikj} \sim \text{Normal}(0,1), j = 1,2, \dots ,5$, two categorical variables independently generated from the multinomial distribution $X_{ikj} \sim \text{Multinomial}(1, .3, .3, .4), j = 6,7$ and three binary variables independently generated from Bernoulli distribution $X_{ikj} \sim \text{Bern}(0.5), j = 8,9,10$ for each individual i in cluster k. We generated three treatment groups with unequal sample sizes; the ratio of individuals across treatment groups was 10:6:1, which is similar to the ratio of individuals across the three

treatment groups of the NCDB data. The treatment assignment mechanism follows a random intercept multinomial logistic regression model,

$$\log\frac{P(A_{ik}=1)}{P(A_{ik}=3)} = 2.2 + .1X_{ik1} + .1X_{ik2} + .1X_{ik3} + .5X_{ik4} + .4X_{ik5} + .2X_{ik6} + .4X_{ik7} + .4X_{ik2}^2 + .4X_{ik2}^2X_{ik5} + \tau_k$$

$$\log\frac{P(A_{ik}=2)}{P(A_{ik}=3)} = 1.4 + .1X_{ik1} + .3X_{ik2} + .2X_{ik3} + .2X_{ik4} + .1X_{ik5} + .4X_{ik6} + .5X_{ik7} - .3X_{ik2}X_{ik4} + .7X_{ik2}^2X_{ik4} + \tau_k$$

where $\tau_k \sim \text{Normal}(0,1)$, $k = 1, \dots, K$.

We simulated the true counterfactual survival times from the Weibull distribution,

$$S_{ik}(t) = \exp\left[-\left\{d_{a_j}\exp\left(m_{a_j}(X_{ik})t\right)\right\}^{\eta}\right]$$

where d_{a_j} is the treatment-group-specific parameter, η is the shape parameter and $m_{a_j}(X_{ik})$ represents a generic functional form of covariates on survival time. Using the inverse transform sampling, we generated counterfactual survival times by,

$$T_{ik}(a_j) = \begin{cases} \left[\dfrac{-\log U}{d_{a_j}\exp\left(-3 + .1X_{ik1} + .3X_{ik2} + \sin(\pi X_{ik3}) + .6X_{ik4} + .5X_{ik5} + 1.2X_{ik6} + .3X_{ik2}^2 + .5X_{ik4}X_{ik5} + b_k\right)}\right]^{\frac{1}{\eta}}, \text{if } a_j = 1 \\ \left[\dfrac{-\log U}{d_{a_j}\exp\left(-1 + .4X_{ik1} + 1.2\sin(\pi X_{ik3}) + 0.4X_{ik4} + 0.3X_{ik5} + X_{ik6} + .8X_{ik7} + .7X_{ik1}^2 + .4X_{ik1}X_{ik4} + b_k\right)}\right]^{\frac{1}{\eta}}, \text{if } a_j = 2 \\ \left[\dfrac{-\log U}{d_{a_j}\exp\left(-2 + .4\sin(\pi X_{ik2}) + .9X_{ik3} + .9X_{ik4} + .4X_{ik5} + .4X_{ik6} + .9X_{ik7} + .4X_{ik4}^2 - .3X_{ik2}X_{ik3} + b_k\right)}\right]^{\frac{1}{\eta}}, \text{if } a_j = 3 \end{cases}$$

where $U \sim \text{Unif}(0,1)$ was a random variable following a uniform distribution on the unit interval $[0,1]$, $b_k \sim N(0,4^2)$. $d_{a_j} = \{5000, 800, 1200\}$ for $a_j = 1,2,3$. The parameter η is set to 2 and $\exp(0.7 + 0.5x_{ik1})$ to respectively produce proportional hazards (PH) and nonproportional hazards (nPH). Observed and uncensored survival times are generated as $T_{ik} = \sum_{a_j \in \text{A}} T_{ik}(a_j) I(A_{ik} = a_j)$. Finally, we generate the censoring time C independently from an exponential distribution with the rate parameter selected to induce 60% censoring. The true 5-

year survival probabilities and 5-year RMST under both PH and nPH settings, as well as the true treatment effects, are summarized in Table S2.

Table S2. True survival probabilities, restricted mean survival time (RMST), and treatment effects under both proportional hazards and nonproportional hazards.

		**PH**		**nPH**	
		**5-year survival**	**5-year RMST**	**5-year survival**	**5-year RMST**
**Treatments**	**1**	0.97	4.97 years	0.96	4.93 years
	**2**	0.68	4.37 years	0.69	4.33 years
	**3**	0.85	4.63 years	0.81	4.58 years
**Treatment effects**	**1 vs. 2**	0.29	7.2 months	0.27	7.3 months
	**1 vs. 3**	0.12	4.0 months	0.15	4.3 months
	**2 vs. 3**	-0.17	-3.2 months	-0.12	-3.0 months

S3. Simulation results

Figure S1 and Figure S2 display the biases and RMSE, across 250 data replications, in subgroup treatment effect estimates among 45 subclasses of true generalized propensity scores under both the PH and nPH settings for each of four methods, IPW-riCox, DR-riAH, riGAPH and riAFT-BART. The treatment effects were estimated based on 5-year survival probability. Our proposed method riAFT-BART boasts the smallest biases and RMSE in all three pairwise treatment effects for all sparsity levels of covariate overlap: not only for the centroid region of the generalized propensity score distribution representing individuals at clinical equipoise, but also for individuals at the tails of the distribution, whose inference about treatment effects are typically difficult to assess. Bias and RMST results for treatment effects based on RMST were similar to Figure S1 and Figure S2, and were therefore omitted. Table S3 demonstrates that our proposed method delivered substantially higher accuracy in estimating the treatment effect heterogeneity (TEH) by the significantly smaller PEHE.

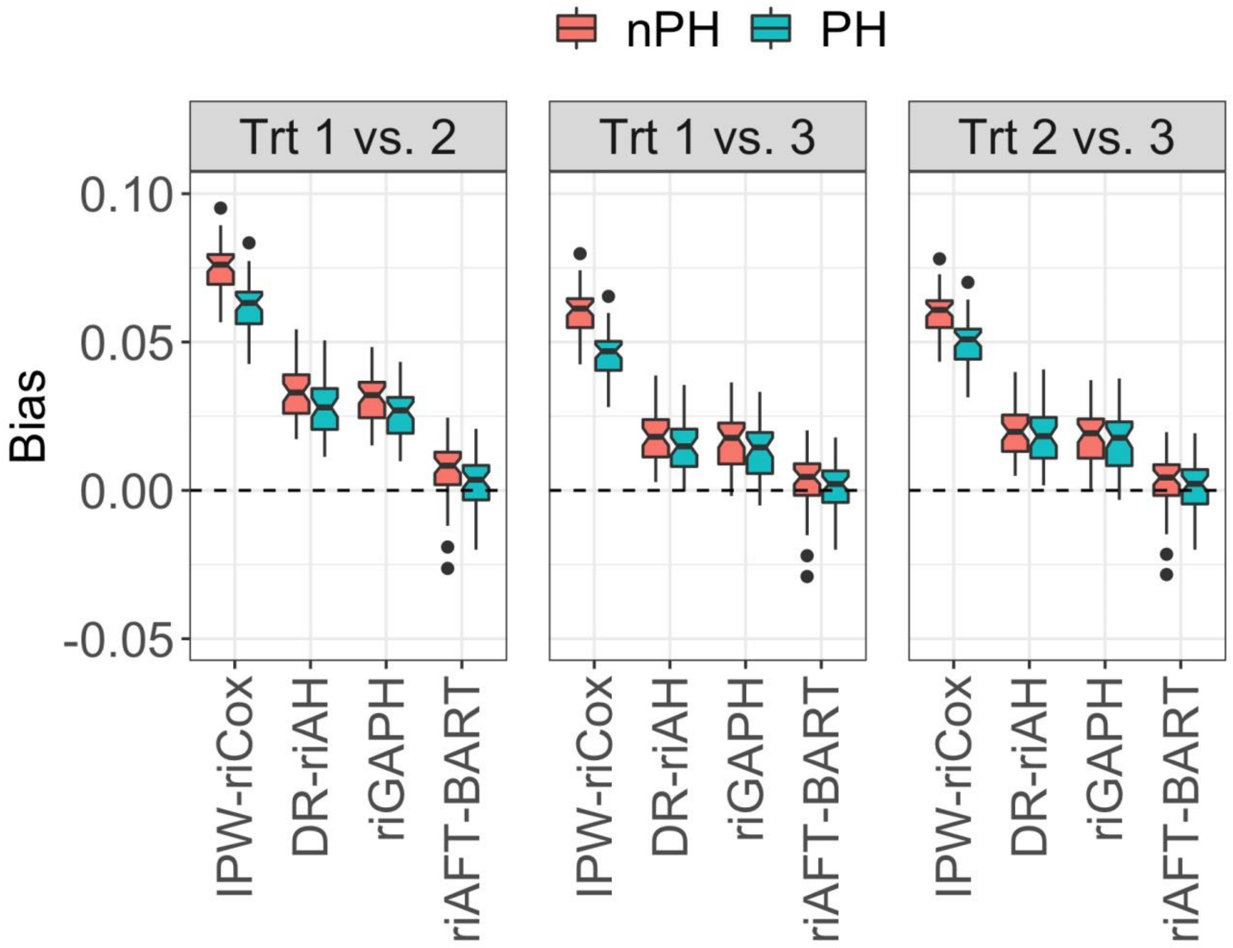

Figure S1. Biases results among subclasses defined by distributions of true generalized propensity scores under proportional hazards (PH) and nonproportional hazards (nPH) for each of four methods, IPW-riCox, DR-riAH, riGAPH and riAFT-BART. Treatment effects were estimated based on 5-year survival probability. Each boxplot visualizes the distribution of biases for 45 subclasses across 250 simulation runs.

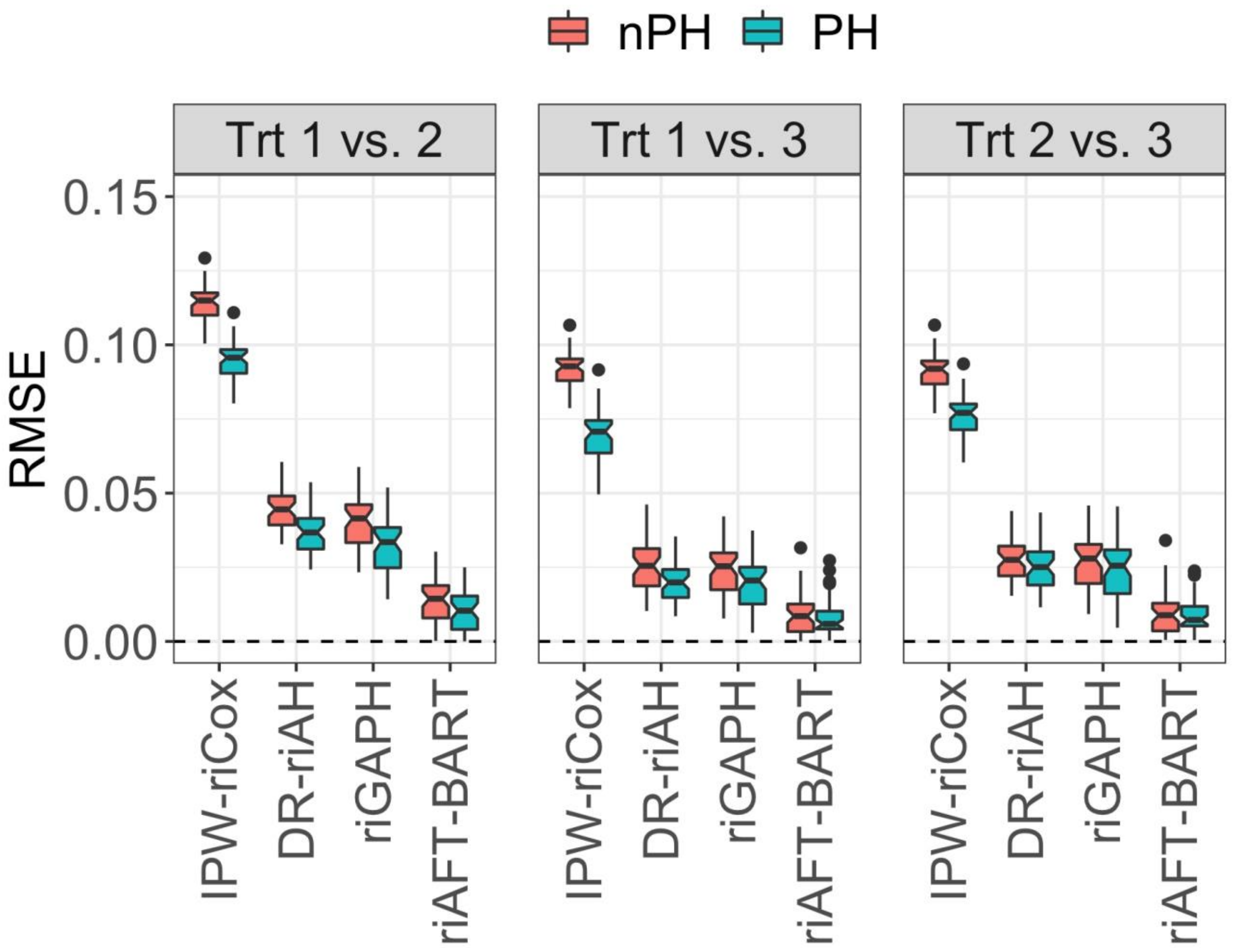

Figure S2. RMSE results among subclasses defined by distributions of true generalized propensity scores under proportional hazards (PH) and nonproportional hazards (nPH) for each of four methods, IPW-riCox, DR-riAH, riGAPH and riAFT-BART. Subgroup treatment effects were estimated based on 5-year survival probability. Each boxplot visualizes the distribution of biases for 45 subclasses across 250 simulation runs.

Table S3. Mean (and standard deviation) of precision in the estimation of heterogeneous effects (PEHE) for each of the 4 methods based on 5-year survival RMST in months.

	**Proportional hazards**			**Nonproportional hazards**		
**Method**	Trt 1 vs. 2	Trt 1 vs. 3	Trt 2 vs. 3	Trt 1 vs. 2	Trt 1 vs. 3	Trt 2 vs. 3
IPW-riCox	2.75 (0.13)	1.99 (0.10)	2.31 (0.12)	3.57 (0.14)	2.63 (0.12)	2.96 (0.13)
DR-riAH	0.76 (0.05)	0.38 (0.04)	0.51 (0.04)	0.93 (0.06)	0.53 (0.04)	0.65 (0.05)
riGAPH	0.74 (0.05)	0.35 (0.04)	0.48 (0.04)	0.90 (0.06)	0.49 (0.04)	0.61 (0.05)
riAFT-BART	0.14 (0.03)	0.10 (0.02)	0.12 (0.02)	0.25 (0.03)	0.21 (0.03)	0.23 (0.03)

S4. Additional results for case study

Figure S3 demonstrates that on average, the expected survival time for patients who underwent RP was 1.25 (1.15, 1.37) times as long as that for patients who underwent EBRT+brachy±AD. However, among high-grade cancer patients with a Gleason score ≥ 9, there is no statistically significant treatment benefit associated with RP. Figure S4 demonstrates that on the population level, when compared to EBRT+AD, RP led to significantly longer survival time; and that patients with higher income ≥ $46,000 and lower Gleason score <9 would experience an enhanced treatment benefit from RP, but there was no directional THE. Figure S5 shows that EBRT+brachy±AD led to a significantly better population patient survival than EBRT+AD; but younger patients (age ≤ 66 years) with lower PSA <11ng/ml had favorable treatment effect from EBRT+AD.

Table S4. Descriptions of pre-treatment variables and hospital locations (clusters) for each of three treatment groups in NCDB data.

	Overall<br>N = 64569	RP<br>N = 37580	EBRT+AD<br>N = 23385	EBRT+brachy±AD<br>N = 3604
Age in years, (mean (SD))	65.61 (8.12)	62.86 (6.98)	69.80 (8.03)	67.07 (7.67)
Race, N (%)				
White	51656 (80.0)	30973 (82.4)	17991 (76.9)	2692 (74.7)

Black	10615 (16.4)	5277 (14.0)	4578 (19.6)	760 (21.1)
American Indian, Aleutian, or Eskimo	2298 (3.6)	1330 (3.5)	816 (3.5)	152 (4.2)
Spanish or Hispanic Origin, N (%)				
Yes	2907 (4.5)	1652 (4.4)	1109 (4.7)	146 (4.1)
No	61662 (95.5)	35928 (95.6)	22276 (95.3)	3458 (95.9)
Insurance, N (%)				
Yes	63264 (98.0)	36840 (98.0)	22863 (97.8)	3561 (98.8)
No	1305 (2.0)	740 (2.0)	522 (2.2)	43 (1.2)
Income, N (%)				
<$30,000	7997 (12.4)	4143 (11.0)	3382 (14.5)	472 (13.1)
$30,000 - $34,999	10961 (17.0)	6060 (16.1)	4285 (18.3)	616 (17.1)
$35,000 - $45,999	17882 (27.7)	10359 (27.6)	6633 (28.4)	890 (24.7)
≥$46,000	27729 (42.9)	17018 (45.3)	9085 (38.8)	1626 (45.1)
Education, N (%)				
<14%	25184 (39.0)	15691 (41.8)	8083 (34.6)	1410 (39.1)
14%-19.9%	15598 (24.2)	9016 (24.0)	5765 (24.7)	817 (22.7)
20% - 28.9%	14150 (21.9)	7798 (20.8)	5557 (23.8)	795 (22.1)
≥29%	9637 (14.9)	5075 (13.5)	3980 (17.0)	582 (16.1)
Clinical T Stage, N (%)				
≤cT2	56522 (87.5)	33856 (90.1)	19601 (83.8)	3065 (85.0)
≥cT3	8047 (12.5)	3724 (9.9)	3784 (16.2)	539 (15.0)
Year of diagnosis, N (%)				
2004-2010	9346 (14.5)	5278 (14.0)	3409 (14.6)	659 (18.3)
2011	10125 (15.7)	5784 (15.4)	3657 (15.6)	684 (19.0)
2012	9662 (15.0)	5656 (15.1)	3467 (14.8)	539 (15.0)
2013	10779 (16.7)	6436 (17.1)	3828 (16.4)	515 (14.3)
2014	11396 (17.6)	6605 (17.6)	4233 (18.1)	558 (15.5)
2015	13261 (20.5)	7821 (20.8)	4791 (20.5)	649 (18.0)

PSA (ng/mL), (mean (SD))	19.93 (21.91)	18.47 (20.68)	22.42 (23.71)	18.93 (20.71)
Gleason score, N (%)				
≤6	4019 (6.2)	3161 (8.4)	676 (2.8)	182 (5.0)
7	11628 (18.0)	6807 (18.1)	4110 (17.6)	711 (19.7)
8	28818 (44.6)	17284 (46.0)	9904 (42.4)	1630 (45.2)
9	18694 (29.0)	9805 (26.1)	7880 (33.7)	1009 (28.0)
10	1410 (2.2)	523 (1.4)	815 (3.5)	72 (2.0)
Location, N (%)				
East North Central	2683 (4.2)	1762 (4.7)	785 (3.4)	136 (3.8)
East South Central	5123 (7.9)	3295 (8.8)	1550 (6.6)	278 (7.7)
Middle Atlantic	9073 (14.1)	5318 (14.2)	3249 (13.9)	506 (14.0)
Mountain	4502 (7.0)	2145 (5.7)	2273 (9.7)	84 (2.3)
New England	12148 (18.8)	6916 (18.4)	4649 (19.9)	583 (16.2)
Pacific	7233 (11.2)	4473 (11.9)	2317 (9.9)	443 (12.3)
South Atlantic	13820 (21.4)	6839 (18.2)	5646 (24.1)	1335 (37.0)
West North Central	6104 (9.5)	4143 (11.0)	1788 (7.6)	173 (4.8)
West South Central	3883 (6.0)	2689 (7.2)	1128 (4.8)	66 (1.8)

Abbreviations: SD = standard deviation; PSA: Prostate-Specific Antigen

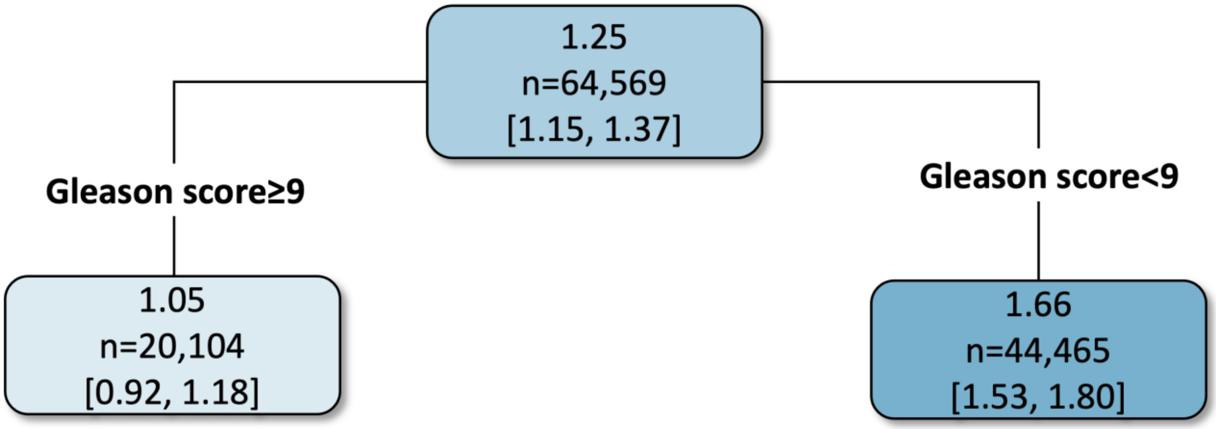

Figure S3. Final Random Forests model fit to the posterior mean of the individual survival treatment effect comparing radical prostatectomy with external beam radiotherapy plus brachytherapy with or without androgen deprivation. Values in each node correspond to the posterior mean and 95% credible intervals of the average treatment effect, in terms of the ratio of expected survival time, for the subgroup of individuals represented in that node.

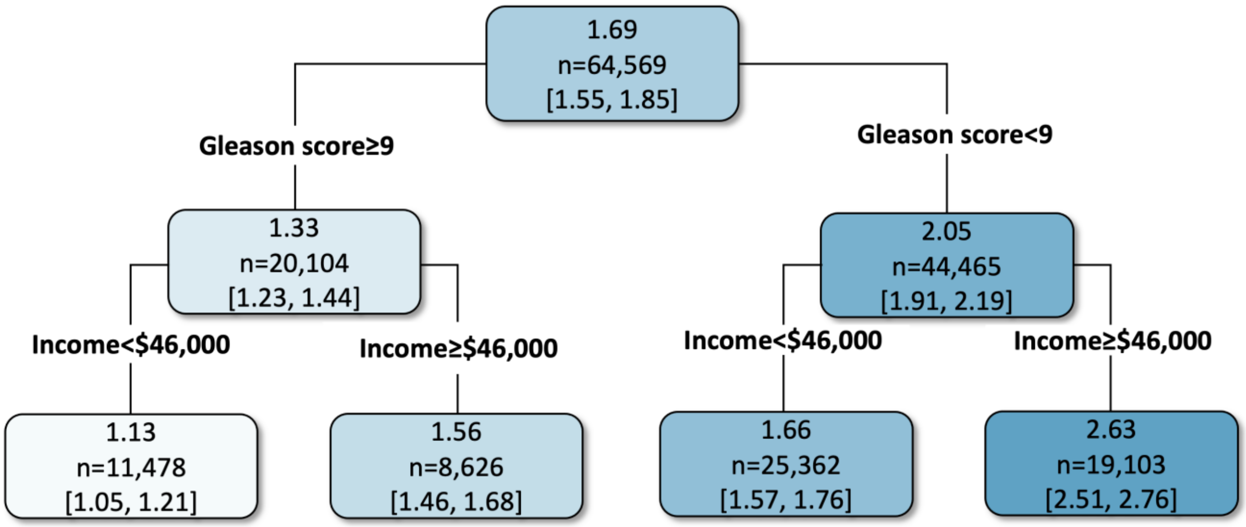

Figure S4. Final Random Forests model fit to the posterior mean of the individual survival treatment effect comparing radical prostatectomy (RP) with external beam radiotherapy combined with androgen deprivation (EBRT+AD). Values in each node correspond to the posterior mean and 95% credible intervals of the average treatment effect, in terms of the ratio of expected survival time, for the subgroup of individuals represented in that node.

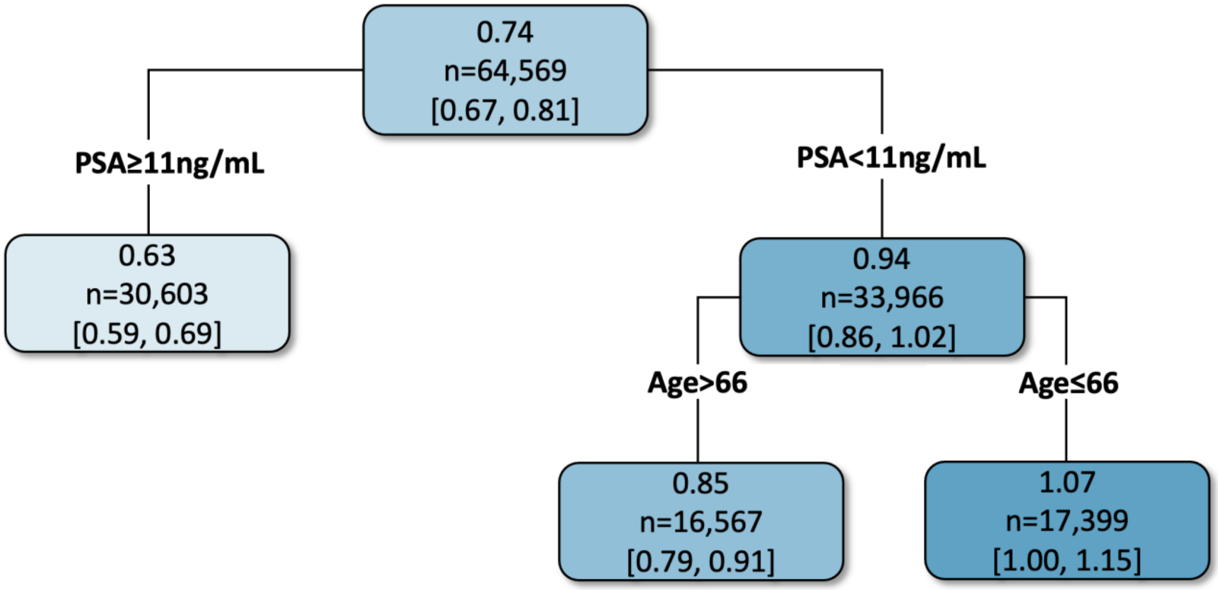

Figure S5. Final Random Forests model fit to the posterior mean of the individual survival treatment effect comparing external beam radiotherapy combined with androgen deprivation (EBRT+AD) to external beam radiotherapy plus brachytherapy with or without androgen deprivation (EBRT+brachy±AD). Values in each node correspond to the posterior mean and 95% credible intervals of the average treatment effect, in terms of the ratio of expected survival time, for the subgroup of individuals represented in that node.

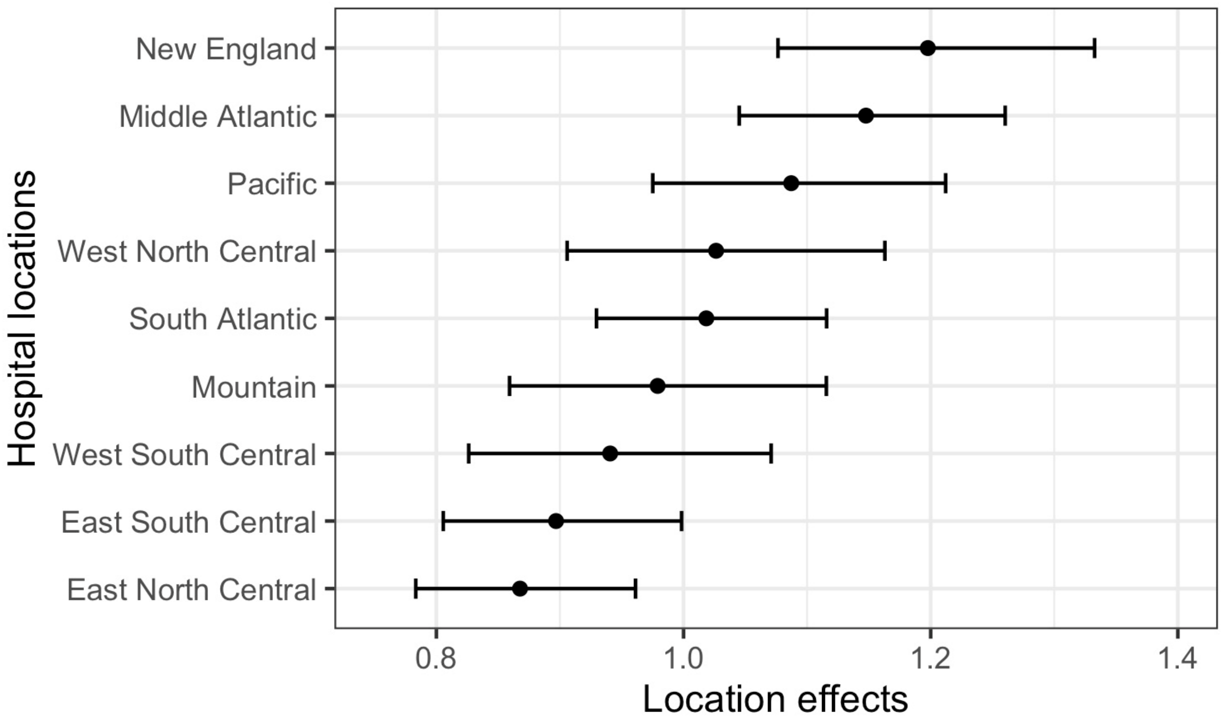

Figure S6. The institutional (location) effects in terms of the expected survival months represented by the posterior mean and credible intervals of random intercept $b_k, k = 1,2, \dots ,9$.

Reference:

1. Hu L, Ji J, Ennis RD, Hogan JW. A flexible approach for causal inference with multiple treatments and clustered survival outcomes. Published online August 10, 2022. doi:10.1002/sim.9548

2. Li F, Zaslavsky AM, Landrum MB. Propensity score weighting with multilevel data. *Stat Med*. 2013;32(19):3373-3387.

3. Hastie TJ, Tibshirani RJ. *Generalized Additive Models*. 1st ed. Boca Raton, FL: Chapman &Hall; 1990.

4. Royston P, Parmar MK. Restricted mean survival time: an alternative to the hazard ratio for the design and analysis of randomized trials with a time-to-event outcome. *BMC Med Res Methodol*. 2013;13(1):152.

5. Hu L, Ji J, Li F. Estimating heterogeneous survival treatment effect in observational data using machine learning. *Stat Med*. 2021;40(21):4691-4713.

6. Lu M, Sadiq S, Feaster DJ, Ishwaran H. Estimating Individual Treatment Effect in Observational Data Using Random Forest Methods. *J Comput Graph Stat*. 2018;27(1):209-219.
